# Interaction between fatty pancreas disease and genetically predicted glucose-dependent insulinotropic polypeptide on incident type 2 diabetes: evidence from the UK Biobank

**DOI:** 10.3389/fendo.2026.1850528

**Published:** 2026-06-08

**Authors:** Xuechen Wang, Yucheng Yang, Shumeng Han, Zijun Liu, Fan Ping, Wei Li, Lingling Xu, Huabing Zhang, Yuxiu Li

**Affiliations:** Department of Endocrinology, Key Laboratory of Endocrinology of National Health Commission, Translation Medicine Center, Peking Union Medical College Hospital, Chinese Academy of Medical Sciences and Peking Union Medical College, Beijing, China

**Keywords:** cohort study, diabetes, ectopic fat, GIP, pancreas fat

## Abstract

**Purpose:**

Glucose-dependent insulinotropic polypeptide (GIP) plays a crucial role in lipid metabolism. The effect of GIP on pancreatic lipid and whether it modulates risk of type 2 diabetes (T2D) associated with fatty pancreas remain unknown. The aim of this study was to investigate the interaction between genetically predicted GIP levels and fatty pancreas in the development of T2D.

**Methods:**

This is a large-scale cohort study using data from the UK Biobank. Participants of White ethnicity without diabetes at the imaging visit were included in the analysis. The loss-of-function GIPR variant E354Q was used for the prediction of fasting GIP levels, and a polygenic risk score (PRS) of postprandial GIP was used for the prediction of postprandial GIP levels. The presence of fatty pancreas disease (FPD) was determined with magnetic resonance imaging (MRI). Diagnosis of T2D was ascertained based on ICD10-CM diagnosis code E11. During a median follow-up of 51 months, 276 cases of incident T2D were identified.

**Results:**

A significant interaction between the carrying status of E354Q and FPD (*p* for interaction = 0.018) and between 2hGIP PRS and FPD (*p* for interaction = 0.015) in the development of T2D was found. FPD is associated with a greater increase of risk of T2D in individuals without E354Q [hazard ratio (HR) 2.44, 95% confidence interval (CI) 1.78–3.34] or with higher levels of genetically predicted postprandial GIP (HR 2.64, 95% CI 1.86–3.76).

**Conclusion:**

Our findings show that genetically predicted GIP modifies risk of T2D associated with FPD, suggesting that GIP may play a role in linking pancreatic fat accumulation to metabolic dysfunction. These findings are derived from genetically predicted rather than measured GIP.

## Introduction

Glucose-dependent insulinotropic polypeptide (GIP) is known for its insulinotropic effects in the pancreas. Beyond the pancreas, expression of GIPR is identified in a wide variety of other tissues, including adipose tissue, bones, cardiomyocytes, and neurons (1). Accumulating evidence suggests that GIP plays a crucial role in lipid metabolism (2). GIP, in the presence of insulin, exerts a lipogenic effect on adipose tissue but stimulates lipolysis under conditions of normo- or hypoinsulinemia (3). Activation of GIPR in adipose tissue protected mice from diet-induced obesity (4). Recent evidence suggests that GIP may also modulate ectopic fat deposition. Higher levels of GIP have been associated with increased visceral fat (5). Additionally, GIP has been suggested to be involved in the pathogenesis of nonalcoholic steatohepatitis (NASH), through increased lipid deposition in liver and adipose tissue inflammation (6). Fatty infiltration of the pancreas, the result of increased fatty acid transport to the pancreas, is also closely associated with metabolic diseases. Similar to subcutaneous and visceral fat, pancreatic adipose tissue was demonstrated to be responsive to GIP (7). However, the role that GIP plays in the lipid metabolism of pancreatic adipocytes remains unknown.

Elevated pancreas fat has been associated with increased risk of type 2 diabetes (T2D). DiRECT (Counterpoint, Counterbalance and the Diabetes Remission Clinical Trial) and other studies have shown that remission of T2D can be achieved through weight loss, and reversal of fatty pancreas is closely associated with remission of T2D (8). Thus, fatty pancreas has emerged as a potential therapeutic target for the prevention and treatment of T2D. In humans, pancreas fat is associated with impaired insulin secretion in individuals with T2D genetic predisposition (9). *In vitro* studies showed that chronic exposure of β cells to fatty acids causes oxidative and ER stress and leads to β cell dysfunction (10, 11). Locally released fatty acids from lipolysis are thus considered to be the key mediators of the detrimental effect of pancreas fat.

In this study, we aimed to investigate the interplay between GIP and fatty pancreas disease (FPD) in the development of T2D using the UK Biobank cohort. We utilized a GIPR loss-of-function variant E354Q, which is associated with lower circulating fasting and postprandial GIP levels and has been utilized as a genetic instrument for fasting GIP levels for the genetic prediction of fasting GIP levels (12). We also generated a genome-wide polygenic risk score (PRS) for circulating postprandial GIP levels for the genetic prediction of postprandial GIP levels. We then examined the interaction between the carrying status of E354Q and FPD and between 2hGIP PRS and FPD in the development of T2D.

## Materials and methods

### Study population

The UK Biobank is a large-scale population-based prospective cohort study. The UK Biobank recruited 9.2 million participants aged 40–69 years between 2006 and 2010. A subset of participants attended the UK Biobank imaging study since 2014 and underwent abdominal magnetic resonance scans. Sociodemographic, lifestyle, and health-related information was collected via a touchscreen questionnaire and verbal interview at the imaging visit. Physical measures were also taken at the imaging visit. Biological samples were collected at recruitment for testing selected biochemical markers and whole-genome genotyping.

In the present study, we included individuals who underwent abdominal magnetic resonance imaging (MRI) scans. Participants with prevalent diabetes at the imaging visit and participants of non-White ethnicity or without genetic data were excluded in the primary analysis.

### Outcomes

The diagnosis of T2D was defined as ICD10-CM diagnosis code E11. Diagnosis information of T2D was ascertained from primary care data, hospital inpatient data, death register records, and self-reported medical conditions.

### Genotyping and PRS

Version 3 of the UK Biobank imputed genotype data, which contains data of approximately 96 million variants, was used. Genotype calling was performed by Affymetrix on two very similar arrays. A total of 49, 950 participants were genotyped on the UK BiLEVE Axiom array, and the remaining 438, 427 participants were genotyped on the UK Biobank Axiom array (13). Genotypes were then imputed using the Haplotype Reference Consortium (HRC) panel and the UK10K panel as the reference panels. Detailed information of the genotyping pipeline, quality control pipeline, and genotype imputation can be found elsewhere (14).

We further generated a PRS for postprandial GIP using single-nucleotide polymorphisms (SNPs) associated with 2hGIP from a GWAS (genome-wide association study) meta-analysis of 7, 828 individuals of European ancestry across the Malmö Diet and Cancer (MDC) and Prevalence, Prediction and Prevention of Diabetes (PPP)–Botnia studies (15). PRS was derived using independent SNPs (*r*^2^ = 0.2 and *w* = 500 kb base pair) associated with 2hGIP at *p* < 5 × 10^−8^ using the clumping + thresholding (C+T) method. A total of four SNPs remained after clumping and thresholding (**Supplementary Table 1**). The limited number of variants retained likely reflects the modest size of the base datasets used for GWAS and may constrain variance explained and the predictive performance of the PRS. For each individual, the 2hGIP PRS was then calculated by aggregating the weighted sum of number of risk alleles at each SNP locus. Participants were categorized into low and high 2hGIP PRS groups by the PRS median. The *F*-statistic and *r*^2^ of E354Q and 2hGIP PRS were calculated for the estimation of variance in the phenotype explained using GWAS summary statistics from Almgren et al. (15). The *F*-statistic and *r*^2^ for E354Q for fasting GIP levels are 57.76 and 0.0073, respectively. The *F*-statistic and *r*^2^ for 2hGIP PRS for postprandial GIP levels are 159.88 and 0.0347, respectively.

### Measurement of pancreas fat

Participants of the UK Biobank imaging study underwent multiecho Dixon acquisition. Pancreas fat was estimated with MRI-derived proton density fat fraction (PDFF), which reflects the concentration of triglycerides within tissues. Estimation of pancreas PDFF was generated using the single-slice multi-echo data with the PRESCO (Phase Regularized Estimation using Smoothing and Constrained Optimization) algorithm.

FPD was defined by the sex- and age-specific 95% upper limit of pancreas PDFF of the general population from the UK Biobank cohort. The cutoff value for the determination of FPD was 8.01% for women aged 45–54 years, 11.03% for women aged 55–64 years, 11.70% for women aged 65 years and older, 11.96% for men aged 45–54 years, 15.77% for men aged 55–64 years, and 12.43% for men aged 65 years and older, as described before (16).

### Covariates

Self-reported ethnic background was acquired via a touchscreen questionnaire at the imaging visit and was grouped into White and non-White ethnic groups. Genetic ethnic grouping was determined with principal components analysis of the genotypes among individuals self-identified as “White British”. Age at the imaging visit was derived based on date of birth and date of reception of the imaging visit. Sex of participants was acquired from central registry at recruitment and in some cases updated by the participants. The Townsend deprivation index (TDI), which is a measure of material deprivation, was calculated prior to recruitment based on national census using the postcodes of participants. Average total household income (<18, 000, 18, 000–30, 999, 31, 000–51, 999, 52, 000–100, 000, and >100 000 £) was acquired via a touchscreen questionnaire at the imaging visit. Qualifications were acquired via a touchscreen questionnaire, and education levels were categorized into college or university degree and others. Drinking status (never, previous, and current) and smoking status (never, previous, and current) were self-reported using a touchscreen questionnaire at the imaging visit. Physical activity information was self-reported via a touchscreen questionnaire, and IPAQ (International Physical Activity Questionnaire) activity group (low, moderate, and high) was derived based on IPAQ guidelines. A healthy diet score (0–5) was calculated based on self-reported frequency of intake of food categories via a touchscreen questionnaire at the imaging visit (vegetable intake ≥ 4 tablespoons/day, fruit intake ≥ 3 pieces/day, fish intake ≥ 2 times/week, unprocessed red meat intake ≤ 2 times/week, and processed meat intake ≤ 2 times/week). Anthropometric measures [including weight, standing height, and body mass index (BMI)] were collected using standard measuring devices at the imaging visit. Selected biochemical markers were measured using the blood sample of participants collected at recruitment.

### Statistical analysis

Baseline characteristics were presented with mean (SD) for continuous variables and counts (proportions) for categorical variables. The Wilcoxon rank sum test and Pearson’s chi-square test were used for comparison of baseline characteristics between groups. Cox proportional hazard models were employed to assess the separate association between FPD and incident T2D and the association between E354Q carrying status or 2hGIP PRS and incident T2D. Schoenfeld residuals were utilized for testing proportional hazard assumptions. To assess effect modification, interaction models where interaction terms were included were generated. Interaction effect was tested using the likelihood ratio test. Missing values for covariates were imputed using the missing indicator approach for categorical covariates and sex-specific mean values for continuous covariates. A sensitivity analysis was conducted where multiple imputations with chained equations were employed for handling missing covariates using the “mice” package of R, version 3.16.0, and the interaction effect was assessed by pooling the Wald test statistics across imputed datasets. The first 10 genetic principal components were included in models for genetic-related analysis. All analyses were performed with R (version 4.3.0).

## Results

### Baseline characteristics

**Figure 1:**
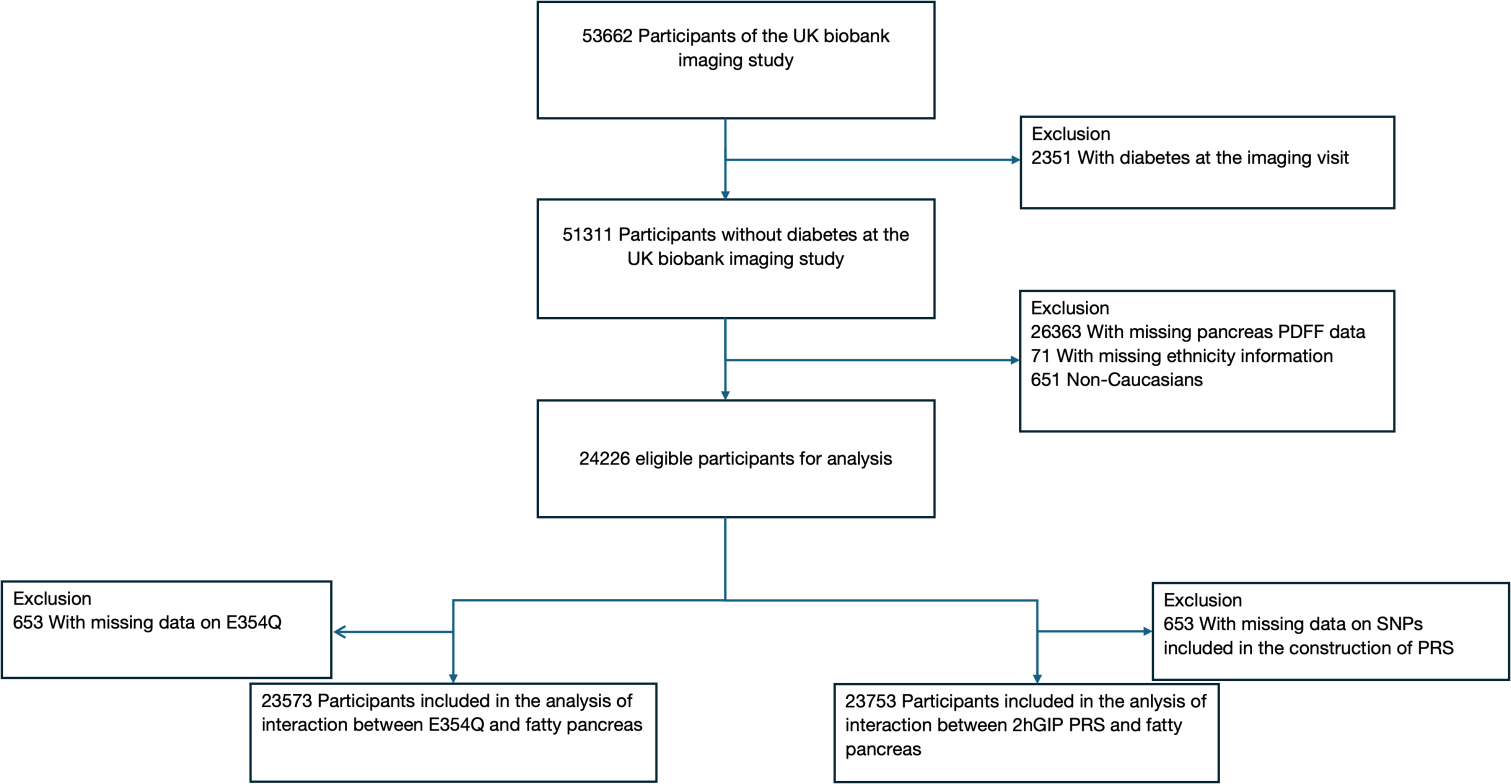
Study participant flow.

A total of 23, 573 participants were included in the primary analysis (**Figure 1**), with a mean age of 64 years. A total of 7, 025 (29.8%) participants were found to have FPD. Participants with FPD were of older age, more likely to be male, more deprived, and less likely to have a college or university degree (**Table 1**). Participants with FPD also had a lower level of physical activity, greater BMI, and a less healthy diet, as can be expected. E354Q (rs180043, C allele) is a missense variant of the GIPR gene. The substitution of G to C and the change from glutamic acid (E) to glutamine (Q) at position 354 lead to impaired GIPR signaling. The proportions of individuals carrying the GIPR variant E354Q and the 2hGIP PRS did not differ between groups. Overall, 3.8% of all participants are homozygous and 31.6% are heterozygous for the E354Q variant, respectively. During a median follow-up of 51 months, 276 cases of incident T2D were identified.

**Table 1:** Baseline characteristics of participants.

Characteristic	No FPD *N* = 16, 548*^1^*	FPD *N* = 7, 025*^1^*	*p*-value*^2^*
Age, years	63 (7)	66 (7)	<0.001
Sex			<0.001
Female	9, 856 (60%)	2, 480 (35%)	
Male	6, 692 (40%)	4, 545 (65%)	
Education			<0.001
College or university degree	8, 689 (53%)	3, 266 (46%)	
Other	7, 859 (47%)	3, 759 (54%)	
Household income, £			<0.001
<18, 000	1, 517 (10%)	808 (13%)	
>100, 000	1, 271 (8.4%)	392 (6.1%)	
18, 000–30, 999	3, 135 (21%)	1, 482 (23%)	
31, 000–51, 999	4, 581 (30%)	1, 982 (31%)	
52, 000–100, 000	4, 641 (31%)	1, 781 (28%)	
thI	−2.02 (2.64)	−1.95 (2.68)	0.043
Smoking status			<0.001
Current	492 (3.0%)	244 (3.5%)	
Never	10, 747 (65%)	4, 008 (58%)	
Previous	5, 174 (32%)	2, 695 (39%)	
Drinking status			0.6
Current	15, 480 (94%)	6, 535 (94%)	
Never	454 (2.8%)	199 (2.9%)	
Previous	518 (3.1%)	235 (3.4%)	
Physical activity			<0.001
High	5, 672 (41%)	2, 255 (38%)	
Low	2, 440 (18%)	1, 172 (20%)	
Moderate	5, 819 (42%)	2, 512 (42%)	
Healthy diet score			<0.001
0	36 (0.2%)	36 (0.5%)	
1	713 (4.4%)	426 (6.3%)	
2	2, 573 (16%)	1, 300 (19%)	
3	4, 576 (28%)	2, 031 (30%)	
4	5, 287 (33%)	2, 000 (29%)	
5	2, 954 (18%)	1, 005 (15%)	
BMI, kg/m^2^	25.4 (3.8)	28.5 (4.2)	<0.001
Pancreas PDFF, %	6 (3)	20 (8)	<0.001
Cholesterol, mmol/L	5.76 (1.05)	5.79 (1.12)	0.026
Triglyceride, mmol/L	1.49 (0.85)	1.92 (1.05)	<0.001
LDL, mmol/L	3.58 (0.81)	3.68 (0.85)	<0.001
HDL, mmol/L	1.54 (0.38)	1.36 (0.33)	<0.001
HbA1c, mmol/mol	34.2 (3.7)	35.1 (4.0)	<0.001
rs1800437			>0.9
C/C	628 (3.8%)	269 (3.8%)	
G/C	5, 222 (32%)	2, 217 (32%)	
G/G	10, 698 (65%)	4, 539 (65%)	
PRS	0.01 (0.08)	0.01 (0.08)	0.090

*^1^*Mean (SD); *n* (%).

*^2^*Wilcoxon rank sum test; Pearson’s chi-square test.

TDI, Townsend deprivation index; BMI, body mass index; PDFF, proton density fat fraction; LDL, low-density lipoprotein; HDL, low-density lipoprotein; HbA1c, hemoglobin A1C.

### Separate effect of FPD, E354Q, and 2hGIP PRS on risk of T2D

A separate effect of FPD E354Q carrying status and 2hGIP PRS on risk of T2D was assessed with Cox proportional hazard models. FPD is associated with an increased risk of T2D adjusted for multiple covariates. Participants with FPD had a 33% higher risk of incident T2D [hazard ratio (HR) 1.33, 95% confidence interval (CI) 1.02–1.73, *p* = 0.034] compared with participants without FPD in the fully adjusted model, consistent with previous findings (**Supplementary Table 2**). Participants without E354Q had a similar risk of T2D (HR 0.93, 95% CI 0.72–1.19, *p* = 0.561) compared with individuals carrying at least one E354Q allele (**Supplementary Table 3**). Similarly, a high PRS of 2hGIP was not significantly associated with increased risk of incident T2D (HR 0.89, 95% CI 0.70–1.14, *p* = 0.362) adjusted for multiple covariates (**Supplementary Table 4**).

### Interaction between genetically predicted GIP and FPD

We then generated interaction models to test for multiplicative interactions between the carrying status of E354Q and the presence of FPD and between 2hGIP PRS and FPD, separately. A significant interaction was identified between the carrying status of E354Q and FPD (*p* for interaction = 0.018), adjusted for multiple covariates (**Table 2**). In participants carrying at least one allele of E354Q, the presence of FPD is associated with a less profound increase of risk of T2D (HR 1.33, 95% CI 0.88–2.00), while in participants without E354Q, FPD is associated with a greater increase of risk of T2D (HR 2.44, 95% CI 1.78–3.34) (**Table 2**). The interaction remained when further adjusted for BMI (*p* for interaction = 0.032), and in participants without E354Q, the presence of FPD is associated with a greater increase of risk of T2D compared with participants carrying E354Q (**Table 2**). Adjustment of BMI attenuated and reversed the association between FPD and T2D in participants carrying E354Q (HR 0.93, 95% CI 0.61–1.42), which could be explained by potential collider bias introduced by controlling for BMI since both E354Q and FPD were associated with BMI levels in previous reports (17). Likewise, a significant multiplicative interaction was found between 2hGIP PRS and FPD on incident T2D (*p* for interaction = 0.015) and in the model further adjusted for BMI (*p* for interaction = 0.027) (**Table 3**). A more profound increase of risk of T2D was found in participants with a high PRS of 2hGIP in the model adjusted for multiple covariates (HR 2.64, 95% CI 1.86–3.76) compared with participants with a low PRS (HR 1.45, 95% CI 1.02–2.06) (**Table 3**).

**Table 2:** Interaction between E354Q and FPD on incident T2D.

Group	C allele carriers		G/G		*P* for interaction
Model 1	HR	CI	HR	CI	0.018
No PFD	Ref	Ref	Ref	Ref	
PFD	1.33	0.88–2.00	2.44	1.78–3.34	
Model 2					0.032
No PFD	Ref	Ref	Ref	Ref	
PFD	0.93	0.61–1.42	1.63	1.18–2.26	

Model 1 was adjusted for age, sex, Townsend deprivation index, household income, education level, drinking status, smoking status, physical activity, healthy diet score, HbA1c, LDL, HDL, total cholesterol, triglycerides, and principal components 1–10. Model 2 was further adjusted for BMI. Abbreviations: HR, hazard ratio; CI, confidence interval.

**Table 3:** Interaction between 2hGIP PRS and FPD on incident T2D.

Group	Low PRS		High PRS		*P* for interaction
Model 1	HR	CI	HR	CI	0.015
No PFD	Ref	Ref	Ref	Ref	
PFD	1.45	1.02–2.06	2.64	1.86–3.76	
Model 2					0.027
No PFD	Ref	Ref	Ref	Ref	
PFD	1.01	0.71–1.45	1.63	1.23–2.55	

Model 1 was adjusted for age, sex, Townsend deprivation index, household income, education level, drinking status, smoking status, physical activity, healthy diet score, HbA1c, LDL, HDL, total cholesterol, triglycerides, and principal components 1–10. Model 2 was further adjusted for BMI. Abbreviations: HR, hazard ratio; CI, confidence interval.

We then examined the interaction effect between pancreas fat and E354Q or 2hGIP PRS. A significant interaction effect was found between 2hGIP PRS and pancreas fat (*p* for interaction = 0.026) (**Table 4**). Per-SD increase of pancreas fat was associated with a 47% higher risk of T2D in individuals with a high 2hGIP PRS (HR 1.47, 95% CI 1.31–1.65) and a 21% higher risk of T2D in individuals with a low 2hGIP PRS (HR 1.21, 95% CI 1.05–1.39) (**Table 4**). Test for interaction did not reach statistical significance between pancreas fat and carrying status of E354Q (*p* for interaction = 0.153). Per-SD increase of pancreas fat was associated with a 24% higher risk of T2D in individuals with carrying E354Q (HR 1.24, 95% CI 1.06–1.44) and a 41% higher risk of T2D in individuals without E354Q (HR 1.41, 95% CI 1.27–1.58) (**Table 5**). The insignificant interaction effect between E354Q carrying status and per-SD increase of pancreas fat suggests that the effect modification of E354Q may be confined to a relatively high level of pancreas fat, though this remains speculative.

**Table 4:** Interaction between 2hGIP PRS and pancreas fat on incident T2D.

1−SD pancreas PDFF	Low PRS		High PRS		*P* for interaction
Model 1	HR	CI	HR	CI	0.026
	1.21	1.05–1.39	1.47	1.31–1.65	
Model 2					0.027
	1.04	0.89–1.22	1.29	1.14–1.48	

Model 1 was adjusted for age, sex, Townsend deprivation index, household income, education level, drinking status, smoking status, physical activity, healthy diet score, HbA1c, LDL, HDL, total cholesterol, triglycerides, and principal components 1–10. Model 2 was further adjusted for BMI. HR, hazard ratio; CI, confidence interval.

**Table 5:** Interaction between E354Q and pancreas fat on incident T2D.

1−SD pancreas PDFF	C allele carriers		G/G		*P* for interaction
Model 1	HR	CI	HR	CI	0.153
	1.24	1.06–1.44	1.41	1.27–1.58	
Model 2					0.181
	1.07	0.90–1.28	1.23	1.09–1.39	

Model 1 was adjusted for age, sex, Townsend deprivation index, household income, education level, drinking status, smoking status, physical activity, healthy diet score, HbA1c, LDL, HDL, total cholesterol, triglycerides, and principal components 1–10. Model 2 was further adjusted for BMI. Abbreviations: HR, hazard ratio; CI, confidence interval.

We further assessed the additive interaction between genetically predicted GIP and FPD in the development of T2D using relative excess risk due to interaction (RERI) and attributable proportion (AP) due to interaction. No significant additive interaction between either the carrying status of E354Q or 2hGIP PRS and FPD on incident T2D was found (**Supplementary Table 5**).

To examine whether the interaction effect of genetically predicted GIP on incident T2D is unique to pancreas fat, we tested the interaction effect between genetically predicted GIP and visceral fat, liver fat, and muscle fat infiltration on the development of T2D separately. No significant interaction effect was found between genetically predicted GIP and visceral fat, liver fat, or muscle fat infiltration (**Supplementary Table 8**).

### Sensitivity analysis

We then conducted sensitivity analysis where the analysis was restricted to participants genetically determined to be Caucasians based on the UK Biobank genetic ethnic grouping data (*n* = 21, 247). Similar to the primary analysis, a significant interaction was found between E354Q carrying status and FPD (*p* for interaction = 0.038), and between 2hGIP PRS and FPD (*p* for interaction = 0.016). The presence of FPD is associated with a greater increase of risk of T2D in individuals without E354Q compared with individuals carrying E354Q and in individuals with a high 2hGIP PRS compared with individuals with a low 2hGIP PRS (**Supplementary Table 6**).

Another sensitivity analysis was conducted using multiple imputation for handling missing covariates. The main results persist when multiple imputation with chained equations was applied. A significant interaction was found between E354Q carrying status and FPD (*p* for interaction = 0.026), and between 2hGIP PRS and FPD (*p* for interaction = 0.021) on incident T2D (**Supplementary Table 7**).

## Discussion

In this large-scale cohort study, we found that genetically predicted fasting and postprandial GIP modified the effect of FPD on incident T2D. The presence of FPD is associated with a more profound increase of risk of T2D in individuals without the GIPR missense variant E354Q and in individuals with a high 2hGIP PRS.

The E354Q variant, a common GIPR variant, is associated with the long-term functional impairment of GIPR. Pharmacological phenotyping revealed that GIPR (E354Q) displayed altered ligand-binding kinetics. GIPR (E354Q) had a prolonged agonist residence time and enhanced agonist-induced receptor internalization (18). Another study showed that *in vitro* stimulation of GIP leads to downregulation of GIPR, and the desensitization process is exaggerated with GIPR (E354Q) (19). In humans, E354Q is associated with lower levels of both fasting and postprandial circulating GIP, and has been linked to T2D, a lower BMI, and cardiovascular diseases (17). A higher level of circulating GIP has also been linked to unhealthy body fat composition and increased carotid intima-media thickness in previous reports (20). Here, we show that genetically predicted circulating GIP levels and E354Q carrying status modified the risk of T2D associated with FPD. Our findings showed that FPD poses less risk in individuals with a lower level of genetically predicted GIP levels.

The effect of GIP on adipose tissue has been largely elucidated. GIP mediates both lipid storage and lipid breakdown. In the presence of insulin, GIP was found to enhance lipoprotein lipase activity in adipocytes (21). GIP also stimulates lipolysis through the cAMP pathway *in vitro* (22). Growing evidence now suggests that GIP may modulate ectopic fat deposition. Variants of GIP were found to be associated with visceral fat accumulation (23). Additionally, elevated GIP was associated with liver injury in NASH (24). Adipose tissue-specific knockout of GIPR led to reduced hepatic steatosis in mice (6). The effect of GIP on pancreas fat remains largely unknown. Though FPD has been associated with incident T2D, pancreas lipid does not lead to islet dysfunction *per se*. More evidence suggests that fatty acids derived from lipolysis of pancreas fat can lead to β cell dysfunction (25). Mechanisms of regulations of pancreas fat lipolysis remains unknown. One recent *in vitro* study of human pancreas organoids showed that pancreatic adipocytes expressed GIPR and stimulation with GIP led to increased lipolytic activity, linking GIP to pancreas fat lipolysis (7). In accordance, our findings suggest that elevated GIP potentially enhances the lipotoxic effect of FPD. Further mechanistic studies are required to determine whether GIP enhances pancreas fat lipolysis *in vivo*, leading to increased local free fatty acids and β cell dysfunction.

The GIP system has become an appealing therapeutic target in treating obesity and diabetes. In addition to glycemic control and weight loss effect, Tirzepatide has also been shown to be protective of the progression of metabolic dysfunction-associated steatohepatitis (26). It remains to be further elucidated whether GIPR targeting therapies will have a beneficial effect in patients with FPD. Our findings suggest that the GIP system may possess therapeutic potential in the treatment of FPD and the prevention of subsequent T2D.

This study has several strengths. Firstly, to the best of our knowledge, this is the first cohort study investigating the interaction effect between pancreas fat and GIPR signaling on the development of T2D. Secondly, this study utilized data from a large-scale cohort study where pancreas fat and presence of FPD was determined with MRI.

Our study also has several limitations. Firstly, genetically predicted GIP rather than actual GIP levels were used in this study, which may only reflect a small and heritable effect due to the relatively small numbers of variants utilized for generation of PRS. Further functional studies are required to validate our results. Secondly, the PRS was derived from a relatively small GWAS (*n* = 7, 828), which may limit the precision of the genetic effect estimates and reduce the predictive performance of the PRS. The findings based on the PRS should thus be interpreted with caution, and future genetic and mechanistic studies are required to further validate our findings. Thirdly, E354Q has been associated with multiple phenotypes, including BMI, cardiovascular diseases, and cancer risk, raising the possibility of potential horizontal pleiotropy. Although the variant was selected based on established biological function, a residual pleiotropic effect cannot be entirely excluded. Fourthly, the study population was restricted to individuals of White ethnicity to ensure ancestry similar to that of the original GWAS population for the generation of PRS, which may affect the generalizability of our results. Fifthly, the median follow-up time was 51 months, which may be insufficient to capture later incident cases and may underestimate the interaction effect. Sixthly, mechanistic inferences regarding the interplay between GIP and FPD are limited in this study due to the lack of longitudinal data for analysis of insulin resistance and β cell function. Furthermore, residual confounding, especially from dietary fat composition and postprandial metabolic state, cannot be excluded, since these factors are important determinants of both GIP secretion and pancreatic fat deposition. In addition, pancreas fat was assessed with a single cross-sectional MRI measurement in this study, which does not capture longitudinal changes over follow-up and may attenuate the true interaction effect.

## Conclusion

In conclusion, genetically predicted GIP has a significant interaction effect with fatty pancreas in the development of T2D. Our findings suggest that fatty pancreas poses a greater threat in individuals with high genetically predicted GIP levels in the development of T2D.

## Data Availability

The original contributions presented in the study are included in the article/**Supplementary Material**. Further inquiries can be directed to the corresponding authors.
