# Supplementary material for: Interaction between fatty pancreas disease and genetically predicted glucose-dependent insulinotropic polypeptide on incident type 2 diabetes: evidence from the UK Biobank
Source: Front Endocrinol (Lausanne). 2026 Jun 8;17:1850528. doi: 10.3389/fendo.2026.1850528 (PMC13283794; doi:10.3389/fendo.2026.1850528)
Supplement: Supplementary file 1 [file Table1.docx]

Supplemental materials

Table S1 Selected genetic variants for generation of 2hGIP PRS

|  | EA | OA | rsid | PVALUE | WEIGHT | gene | POS | CHROM |
| --- | --- | --- | --- | --- | --- | --- | --- | --- |
| 1 | A | G | rs17683430 | 3.00E-18 | 0.122 | SLC5A1 | 32091713 | 22 |
| 2 | C | G | rs1800437 | 2.00E-17 | -0.072 | GIPR | 45678134 | 19 |
| 3 | T | C | rs635634 | 7.00E-14 | -0.07 | Y_RNA,ABO | 133279427 | 9 |
| 4 | T | C | rs927332 | 4.00E-08 | 0.04 | F13A1,LY86-AS1 | 6331640 | 6 |

Table S2 Associations between FPD and T2D

| Model1 | HR | CI | P |
| --- | --- | --- | --- |
| No FPD | ref | ref |  |
| FPD | 2.48 | 1.94-3.16 | <0.001 |
| Model 2 |  |  |  |
| No FPD | ref | ref |  |
| FPD | 1.93 | 1.50-2.49 | <0.001 |
| Model 3 |  |  |  |
| No FPD | ref | ref |  |
| FPD | 1.33 | 1.02-1.73 | 0.034 |

Model 1 was adjusted for age and sex. Model 2 was adjusted for age, sex, Townsend deprivation index, household income, education level, drinking status, smoking status, physical activity, healthy diet score, HbA1c, LDL, HDL, total cholesterol, triglycerides. Model 3 was further adjusted for BMI. Abbreviations: HR, hazard ratio; CI, confidence interval.

Table S3 Associations between E354Q and T2D

| Model 1 | HR | CI | P |
| --- | --- | --- | --- |
| C allele carriers | ref | ref |  |
| G/G | 0.99 | 0.78-1.27 | 0.966 |
| Model 2 |  |  |  |
| C allele carriers | ref | ref |  |
| G/G | 0.99 | 0.77-1.27 | 0.939 |
| Model 3 |  |  |  |
| C allele carriers | ref | ref |  |
| G/G | 0.93 | 0.72-1.19 | 0.561 |

Model 1 was adjusted for age and sex. Model 2 was adjusted for age, sex, Townsend deprivation index, household income, education level, drinking status, smoking status, physical activity, healthy diet score, HbA1c, LDL, HDL, total cholesterol, triglycerides, principal components 1-10. Model 3 was further adjusted for BMI. Abbreviations: HR, hazard ratio; CI, confidence interval.

Table S4 Associations between 2hGIP PRS and T2D

| Model 1 | HR | CI | P |
| --- | --- | --- | --- |
| Low PRS | ref | ref |  |
| High PRS | 1.01 | 0.80-1.28 | 0.935 |
| Model 2 |  |  |  |
| Low PRS | ref | ref |  |
| High PRS | 0.94 | 0.74-1.20 | 0.620 |
| Model 3 |  |  |  |
| Low PRS | ref | ref |  |
| High PRS | 0.89 | 0.70-1.14 | 0.362 |

Model 1 was adjusted for age and sex. Model 2 was adjusted for age, sex, Townsend deprivation index, household income, education level, drinking status, smoking status, physical activity, healthy diet score, HbA1c, LDL, HDL, total cholesterol, triglycerides, principal components 1-10. Model 3 was further adjusted for BMI. Abbreviations: HR, hazard ratio; CI, confidence interval.

Table S5 Additive interaction between genetically predicted GIP and FPD on incident T2D

|  | RERI (95% CI) | AP (95% CI) |
| --- | --- | --- |
| E354Q carrying status | -0.73 (-1.5, 0.04) | -0.54 (-1.17, 0.09) |
| 2hGIP PRS | -0.75 (-1.56, 0.06) | -0.49 (-1.04, 0.06) |

Model was adjusted for age, sex, Townsend deprivation index, household income, education level, drinking status, smoking status, physical activity, healthy diet score, HbA1c, LDL, HDL, total cholesterol, triglycerides, BMI, principal components 1-10. Abbreviations: CI, confidence interval; RERI, relative excess risk due to interaction; AP, attributable proportion.

Table S6 Sensitivity analysis restricted to genetically determined Caucasians

|  | C allele carriers | | G/G | | P for interaction |
| --- | --- | --- | --- | --- | --- |
| Model 1 | HR | CI | HR | CI | 0.038 |
| No PFD | ref | ref | ref | ref |  |
| PFD | 1.26 | 0.81-1.95 | 2.22 | 1.59-3.09 |  |
| Model 2 |  |  |  |  | 0.057 |
| No PFD | ref | ref | ref | ref |  |
| PFD | 0.90 | 0.58-1.41 | 1.52 | 1.08-2.14 |  |
|  | Low PRS | | High PRS | | P for interaction |
| Model 1 | HR | CI | HR | CI | 0.016 |
| No PFD | ref | ref | ref | ref |  |
| PFD | 1.29 | 0.89-1.89 | 2.50 | 1.72-3.63 |  |
| Model 2 |  |  |  |  | 0.036 |
| No PFD | ref | ref | ref | ref |  |
| PFD | 0.91 | 0.61-1.34 | 1.72 | 1.18-2.52 |  |

Model 1 was adjusted for age, sex, Townsend deprivation index, household income, education level, drinking status, smoking status, physical activity, healthy diet score, HbA1c, LDL, HDL, total cholesterol, triglycerides, principal components 1-10. Model 2 was further adjusted for BMI. Abbreviations: HR, hazard ratio; CI, confidence interval.

Table S7 Sensitivity analysis using multiple imputation

|  | C allele carriers | | G/G | | P for interaction |
| --- | --- | --- | --- | --- | --- |
| Model 1 | HR | CI | HR | CI | 0.026 |
| No PFD | ref | ref | ref | ref |  |
| PFD | 1.30 | 0.86-1.97 | 2.33 | 1.70-3.19 |  |
| Model 2 |  |  |  |  | 0.047 |
| No PFD | ref | ref | ref | ref |  |
| PFD | 0.99 | 0.65-1.50 | 1.67 | 1.20-2.31 |  |
|  | Low PRS | | High PRS | | P for interaction |
| Model 1 | HR | CI | HR | CI | 0.021 |
| No PFD | ref | ref | ref | ref |  |
| PFD | 1.41 | 0.99-2.01 | 2.52 | 1.77-3.61 |  |
| Model 2 |  |  |  |  | 0.027 |
| No PFD | ref | ref | ref | ref |  |
| PFD | 1.05 | 0.73-1.50 | 1.82 | 1.27-2.62 |  |

Model 1 was adjusted for age, sex, Townsend deprivation index, household income, education level, drinking status, smoking status, physical activity, healthy diet score, HbA1c, LDL, HDL, total cholesterol, triglycerides, principal components 1-10. Model 2 was further adjusted for BMI. Abbreviations: HR, hazard ratio; CI, confidence interval.

Table S8 Interaction between genetically predicted GIP and liver fat, muscle fat infiltration and visceral fat on incident T2D

| E354Q | C allele carriers | | G/G | | P for interaction |
| --- | --- | --- | --- | --- | --- |
|  | HR | CI | HR | CI |  |
| Liver PDFF | 1.12 | 1.09-1.15 | 1.11 | 1.09-1.14 | 0.689 |
| Muscle fat infiltration | 1.21 | 1.10-1.32 | 1.20 | 1.14-1.27 | 0.924 |
| Visceral fat | 1.30 | 1.20-1.40 | 1.31 | 1.23-1.39 | 0.874 |
| 2hGIP PRS | Low PRS | | High PRS | | P for interaction |
|  | HR | CI | HR | CI |  |
| Liver PDFF | 1.11 | 1.09-1.14 | 1.11 | 1.08-1.14 | 0.982 |
| Muscle fat infiltration | 1.17 | 1.09-1.26 | 1.23 | 1.15-1.31 | 0.316 |
| Visceral fat | 1.28 | 1.19-1.37 | 1.33 | 1.25-1.42 | 0.315 |

Models were adjusted for age, sex, Townsend deprivation index, household income, education level, drinking status, smoking status, physical activity, healthy diet score, HbA1c, LDL, HDL, total cholesterol, triglycerides, principal components 1-10.
